# Incidence and predictors of complications of tube thoracostomy performed in the emergency department for non-traumatic pleural pathologies

**DOI:** 10.1007/s11739-025-04148-2

**Published:** 2025-10-26

**Authors:** Andrea Rossetto, Martino V. Aiello, Ludovica Tamburrino Orlando, Giada Ermini, Annamaria Cazzolla, Peiman Nazerian

**Affiliations:** 1 University of Florence, Florence, Italy; 2 Department of Emergency Medicine, Careggi University Hospital, Largo Brambilla 3, 50134 Florence, Italy; 3 University of Bari, Bari, Italy

**Keywords:** Tube thoracostomy, Pleural effusion, Pneumothorax, Emergency department

## Abstract

Tube thoracostomy (TT) is a common emergency procedure for the management of non-traumatic pleural conditions in the emergency department (ED). While effective, it carries a significant risk of complications. We aimed to assess the incidence and predictors of complications associated with TT performed in the ED for non-traumatic pleural pathologies. Retrospective cohort study involving adult patients undergoing TT in the ED of a University Hospital between 2020 and 2023 in Italy. Multivariable logistic regression was used to identify independent risk factors for complications. We enrolled 293 adult patients who underwent TT in the ED. Of these, 40% (116/293) were female, and the median age was 70 years (50–78). A pleural effusion was diagnosed in 67% (197/293) of patients, 11% (20/197) of whom had a loculated effusion, whereas a pneumothorax in 43% (126/293). TT-related complications occurred in 24% of cases, with malposition/dislodgment (13%), pneumothorax (9%) and obstruction (5%) most frequent. No deaths were directly attributed to TT. Multivariable analysis identified higher platelet counts (OR 1.39, 95%CI 1.02–1.89, p = 0.035) and localized pleural disease (OR 1.44, 95%CI 1.09–1.90, p = 0.011) as independent risk factors. No significant association was found with operator specialty, seniority, drain type, or time of procedure. TT performed in the ED for non-traumatic pleural diseases is associated with a complication rate comparable to other settings. Complicated pleural pathology and elevated platelet count were the main predictors of adverse events.

## Introduction

Tube thoracostomy (TT) is one of the most common surgical procedures performed in clinical practice, that can be executed at the patient's bedside, in the operating room or in the emergency department (ED), sometimes in life-threatening situations [1]. A thoracostomy tube is inserted to maintain negative intra-thoracic pressures by evacuating air, blood or fluids retained in the pleural cavity, promoting lung re-expansion, improving respiratory function and facilitating postoperative recovery in patients undergoing thoracic/cardiac surgery [1]. Despite its many advantages, TT can be a significant iatrogenic source of morbidity with overall reported complication rates of up to 30–37% leading to increased length of hospital stay and related hospitalization costs [2]. Among the most common complications related to TT, there are the malposition, dislodgment or obstruction of the tube, iatrogenic pneumothorax (PNX), organ damage, haemorrhages and infections. [3]

Various risk factors for the development of TT complications have been identified by previous studies, such as the use of chest tubes with a diameter smaller than 36 mm, [2] placement in the ED, elevated body mass index [4], lack of adequate supervision and out-of-hour procedures [5]. There is instead conflicting evidence in relation to the specialist preforming the procedure, with some evidence reporting increased risk of TT complications following the placement of a chest tube by emergency physicians, while another study found no difference in the rate of complications when the procedure was performed by either thoracic surgery or emergency medicine residents [4, 6]. The procedure can be usually performed in fact by thoracic surgeons, as well as emergency physicians, intensivists, pulmonologists and interventional radiologists. Although previous studies describe complications from pleural drainage in various hospital settings performed by physicians from different specialties [1], TT complications following placement specifically in the ED are not widely described in the literature [4]. Moreover, many studies investigate the complication rate of TT specifically in trauma patients, with limited evidence on non-traumatic pleural pathologies. [7]

The purpose of this investigation was to evaluate the incidence and main risk factors associated with TT when performed in the ED for non-traumatic pleural pathologies by either emergency medicine (EM) or thoracic surgery physicians.

## Methods

### Study design

We performed an observational retrospective cohort study involving patients admitted to the ED of a University Hospital in Florence, Italy, between January 2020 and January 2023 who underwent tube thoracostomy for any non-traumatic cause, namely spontaneous pneumothorax and pleural effusion. Exclusion criteria were age < 18 years, TT performed at an outside facility, trauma-related TT and evacuation of an emo/pneumothorax by percutaneous catheter aspiration or drainage without placement of a thoracostomy tube. Patients who underwent multiple TT in subsequent admissions in the ED were considered as independent procedures if the prior TT was purposefully removed. The study was approved by the local Ethics Committee (27001_oss, 23/07/2024) and complied with the Declaration of Helsinki and good clinical practice guidelines.

### Data collection

The following variables were collected from patients' electronic medical records: age, sex, triage code, primary complaint, past medical history, vital signs, laboratory parameters and the presence of complex anatomy, defined as the presence of hepatomegaly, splenomegaly, cardiomegaly, elevated diaphragm or skeleton deformities. We also collected data regarding the diagnosis for which TT was performed, type of tube inserted, the use of ultrasound guidance, whether the procedure was performed overnight (22:00–8:00) and the operator performing the TT, at our institution primarily either thoracic surgery consultants (TS-C) or residents (TS-R) or emergency medicine consultants (EM-C) or residents (EM-R). Residents were always supervised by consultant physicians when performing TT. As outcomes, we included whether or not TT complications developed, the type of complications (malposition/displacement, obstruction, PNX, haemothorax, empyema, organ injury, major vascular injury, skin infection and hemorrhage), disposition, length of stay and mortality.

### Statistical analysis

Continuous variables were reported as median and interquartile range (IQR), with statistical significance assessed using the Mann–Whitney U test or the Kruskal–Wallis test. Categorical variables were reported as frequency and percentage, with statistical significance evaluated using Fisher's exact test for binary variables and Pearson's chi-square test for variables with more than two categories. Bonferroni correction was applied in multiple comparisons between subgroups. To assess the effect of risk factors on the development of post-TT complications, a multivariable logistic regression analysis was performed. The following variables were included in the model: age, sex, platelet count, pulmonary neoplastic disease, loculated effusion/pneumothorax, operator specialty and level of expertise, tube type, and whether the procedure was performed during night-time hours (22:00–08:00). All variables were included in the multivariable regressions independently of their statistical significance in the univariable analysis [8]. Variables were normalized by subtracting the mean and dividing by the standard deviation prior to their inclusion in the regressions to facilitate the interpretation of their relative independent effects. Multicollinearity was quantified using the variance inflation factor (VIF). A complete case analysis was conducted, and a significance level of 0.05 was used. Statistical analyses were performed using RStudio 1.2.1335 (RStudio Inc., MA, USA).

## Results

### Study cohort and incidence of TT complications

This study analysed data from 293 adult patients who underwent TT in the ED between January 2020 and January 2023. Of these, 40% (116/293) were female, and the median age was 70 years (50–78) (Table 1). Most patients were admitted to the ED for dyspnoea (220/293, 75%). Pulmonary neoplasia, either primary or secondary, was present in 37% (109/293) of patients, while nearly a quarter (65/293) had other non-neoplastic pulmonary conditions, most commonly COPD (37/65, 57%). A pleural effusion was diagnosed in 67% (197/293) of patients, 11% (20/197) of whom had a loculated effusion, whereas a pneumothorax in 43% (126/293) (Table 2). Thirty-one patients (11%) presented with both conditions. At least one TT-related complication developed in 24% (71/293) of patients; however, no patient in the cohort died due to a procedure-related complication. The most frequent complications were malposition/dislodgment (13%), obstruction (5%) and iatrogenic pneumothorax (9%) (Table 3).

**Table 1:** Cohort characteristics

	Overall	No Complications	Complications	
	N = 293	N = 222	N = 71	p
Demographics and triage				
Age, years	70 (50–78)	69 (48–78)	71 (63–79)	0.076
Sex, female	116 (40%)	81 (36%)	35 (49%)	0.055
Triage code				0.535
1 – Resuscitation	6 (2%)	4 (2%)	2 (3%)	
2 – Emergent	69 (24%)	49 (22%)	20 (28%)	
3 – Urgent	215 (73%)	166 (75%)	49 (69%)	
4 – Less Urgent	3 (1.0%)	3 (1.4%)	0 (0%)	
Chief complaint				0.617
Dyspnea	220 (75%)	164 (74%)	56 (79%)	
Pain	48 (16%)	39 (18%)	9 (13%)	
Other	25 (9%)	19 (9%)	6 (9%)	
Past medical history				
Lung disease	65 (22%)	54 (24%)	11 (15%)	0.119
Lung neoplasm	109 (37%)	74 (33%)	35 (49%)	**0.015**
Previous lung surgery	35 (12%)	25 (11%)	10 (14%)	0.523
Previous chest tube	89 (30%)	71 (32%)	18 (25%)	0.281
Complex anatomy	35 (12%)	26 (12%)	9 (13%)	0.827
Comorbidities	91 (31%)	67 (30%)	24 (34%)	0.581
A/P or A/C therapy	93 (32%)	69 (31%)	24 (34%)	0.668
Vitals and laboratory parameters				
Systolic blood pressure, mmHg	132 (117–149)	130 (117–147)	140 (120–150)	0.248
PaO_2_/FiO_2_	314 (261–355)	314 (257–352)	329 (270–365)	0.196
Lactate, mEq/L	1.2 (0.7–2.0)	1.3 (0.6–2.1)	1.1 (0.7–1.9)	0.567
White blood cell count, × 10^6^/mL	9.6 (7.3–13.4)	9.5 (7.3–12.2)	9.8 (7.3–16.4)	0.293
C-reactive protein, mg/dL	32 (9–91)	30 (7–84)	58 (19–113)	0.085
Platelet count, × 10^6^/mL	269 (212–352)	256 (202–337)	317 (263–388)	** < 0.001**
International normalized ratio	1.1 (1.1–1.3)	1.1 (1.1–1.3)	1.1 (1.1–1.2)	0.928
Creatinine, mg/dL	0.85 (0.68–1.06)	0.85 (0.69–1.06)	0.82 (0.67–1.01)	0.479
Clinical outcomes				
Mortality due to TT complications	0 (0%)	0 (0%)	0 (0%)	> 0.999
Length of stay, days	6 (2–12)	5 (2–11)	9 (6–17)	** < 0.001**
ED outcome				**0.013**
ED mortality	3 (1.0%)	2 (0.9%)	1 (1.4%)	
ICU admission	9 (3%)	7 (3%)	2 (3%)	
Ward admission	190 (65%)	134 (60%)	56 (79%)	
Discharge	91 (31%)	79 (36%)	12 (17%)	
Hospital outcome				0.731
In-hospital mortality	23 (12%)	17 (12%)	6 (10%)	
Discharge	176 (88%)	124 (88%)	52 (90%)	

Dasa reported as median (interquartile range) or frequency (%). *TT* tube thoracostomy, *ED* emergency department

**Table 2:** Diagnostics and procedure characteristics

	Overall	No complications	Complications	
	N = 293	N = 222	N = 71	p
Diagnostics				
Radiography	253 (86%)	194 (87%)	59 (83%)	0.360
Ultrasound	179 (61%)	125 (56%)	54 (76%)	**0.003**
Computed tomography	60 (20%)	42 (19%)	18 (25%)	0.242
Pneumothorax	126 (43%)	112 (50%)	14 (20%)	** < 0.001**
Localised	4 (4%)	3 (3%)	1 (9%)	0.353
Pleural effusion	197 (67%)	135 (61%)	62 (87%)	** < 0.001**
Localised	20 (11%)	10 (8%)	10 (16%)	0.080
Overnight procedure	42 (14%)	30 (14%)	12 (17%)	0.487
Procedure characteristics				
Clinician				0.381
EM consultant	120 (41%)	89 (41%)	31 (44%)	
EM resident	35 (12%)	24 (11%)	11 (15%)	
Thoracic surgery consultant	91 (31%)	73 (33%)	18 (25%)	
Thoracic surgery resident	42 (14%)	32 (15%)	10 (14%)	
Others consultant	2 (0%)	1 (0%)	1 (0%)	
Tube type				**0.023**
Minimally invasive	202 (76%)	146 (72%)	56 (86%)	
Trocar	65 (24%)	56 (28%)	9 (14%)	
Tube size,Fr	12 (12–20)	12 (12–20)	12 (10–14)	**0.020**
Ultrasound methodology				0.764
Ultrasound-assisted	265 (95%)	201 (95%)	64 (94%)	
Ultrasound-guided	15 (5%)	11 (5%)	4 (6%)	

Data reported as median (interquartile range) or frequency (%). *EM* emergency medicine

**Table 3:** Complications of tube thoracostomy

	N = 293
Malposition/displacement	38 (13%)
Obstruction	16 (5%)
Pneumothorax	27 (9%)
Haemothorax	1 (0%)
Empyema	3 (1%)
Organ injury	5 (2%)
Major vascular injury	0 (0%)
Skin infection	0 (0%)
Skin haemorrhage	0 (0%)

Data reported as frequency (%)

### Clinical and procedural characteristics in patients who developed TT complications

In patients who developed complications, there was a signal for older age (69 years, 48–78, vs 71 years, 63–79; p = 0.076) and a higher prevalence of female sex (81/222, 36% vs. 35/71, 49%; p = 0.055) (Table 1). Additionally, patients with TT-related complications suffered most frequently from pulmonary neoplastic diseases (74/222, 33% vs. 35/71, 49%; p = 0.015). No significant differences were found between groups regarding the number and type of other comorbidities analysed. Among vital signs and laboratory parameters, a significant difference was observed in platelet count (256 × 10⁹, 202–337 vs. 317 × 10⁹, 263–388; p < 0.001), with higher levels in patients who developed complications. Of the patients who experienced complications, 82% (58/71) required hospitalization, with the majority (97%, 56/58) admitted to a general ward and only 3% (2/58) to the intensive care unit and had overall a significantly longer hospital stay (9 days, 6–17 vs. 5 days, 2–11; p < 0.001). Patients who developed at least one complication were more frequently affected by pleural effusion (135/222, 61% vs. 62/71, 87%; p < 0.001), particularly loculated forms (10/222, 8% vs. 10/71, 16%; p = 0.080), rather than pneumothorax (112/222, 50% vs. 14/71, 20%; p < 0.001) (Table 2). Regarding the type of chest drainage inserted, patients who developed complications were more frequently managed with a minimally invasive drainage system (146/222, 72% vs. 56/71, 86%; p = 0.023). The distribution of procedures performed by consultant physicians and residents in emergency medicine or thoracic surgery, respectively, was similar (p = 0.381).

### Clinical and procedural characteristics according to the TT operator

**Fig. 1:**
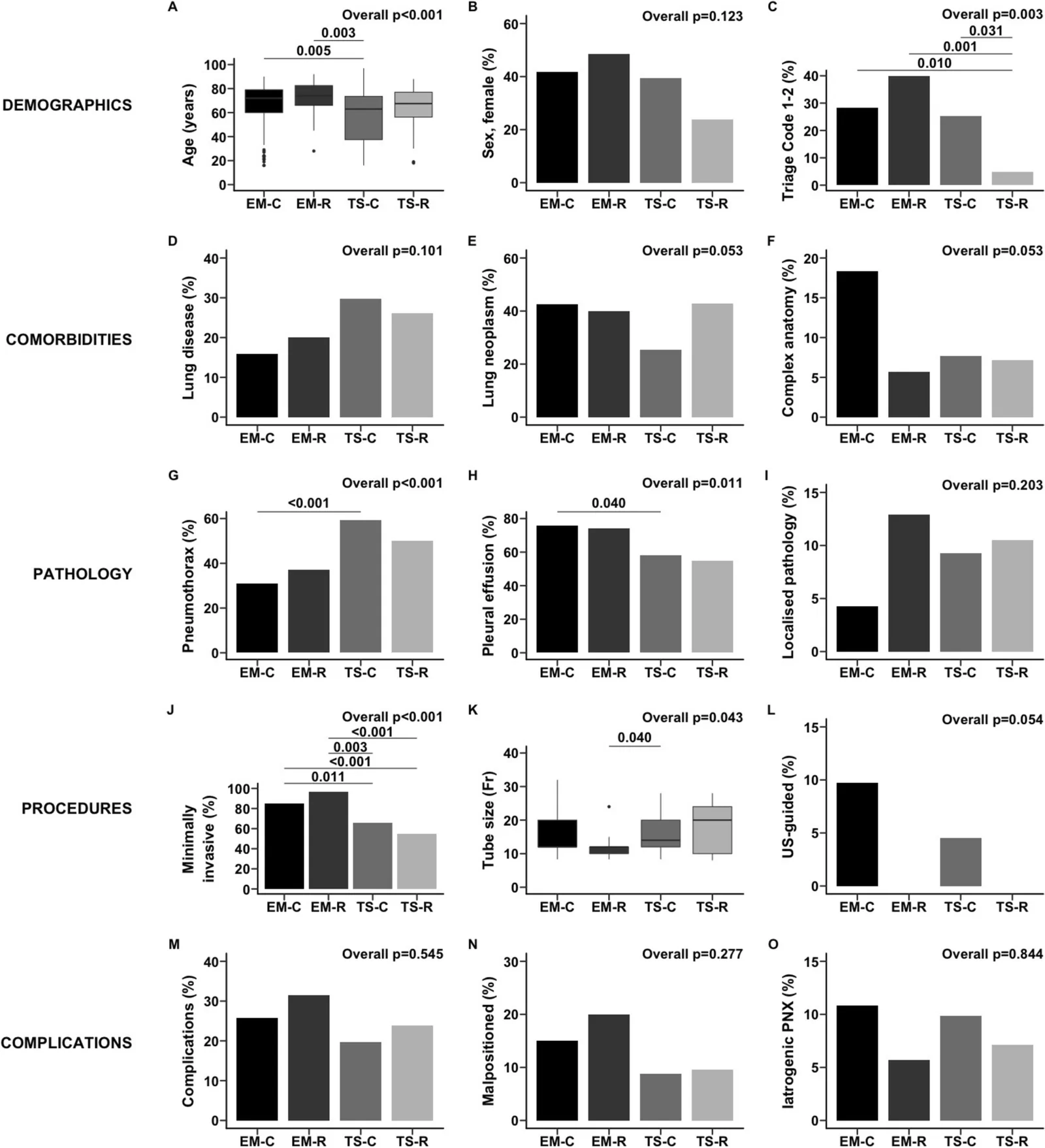
Characteristics of the study cohort according to the clinician performing tube thoracostomy. Kruskal–Wallis and Pearson's chi-square tests were used for overall statistical comparisons, with Bonferroni correction applied to between-subgroup comparisons. *EM-C* emergency medicine consultant, *EM-R* emergency medicine resident, *TS-C* thoracic surgery consultant, *TS-R* thoracic surgery resident

In terms of patients’ demographics, younger patients were more frequently treated by TS-C (p < 0.001; Fig. 1A). On the other hand, in critically ill patients TT was primarily performed by EM-R (triage codes 1–2, p = 0.003; Fig. 1C). A trend was observed indicating that EM-C performed TT in patients with complex anatomy more frequently than other operators (p = 0.053; Fig. 1F). Thoracic surgery practitioners performed more TT in patients presenting with PNX (p < 0.001; Fig. 1G), while those with pleural effusions were primarily treated by EM-C or EM-R (p = 0.011; Fig. 1H). Minimally invasive drains were more commonly used than larger drains by emergency medicine practitioners (p < 0.001; Fig. 1J). However, no significant differences were found in post-TT complication rates according to the performing operator.

### Localised pleural pathology is associated with more TT complications

Multivariable regression analysis identified higher platelet counts (OR 1.39, 95%CI 1.02–1.89; p = 0.035) and localized pleural disease (OR 1.44, 95%CI 1.09–1.90; p = 0.011) as independent risk factors for post-TT complications (Table 4). Other variables considered in the logistic regression model, including operator specialty/training level and drain type, did not show an independent effect on the development of complications.

**Table 4:** Multivariable logistic regression analysis for any tube thoracostomy complication

	OR	(95% CI)	p	Adj. OR	(95% CI)	p
Age	1.42	(1.03—1.97)	**0.034**	1.25	(0.84—1.84)	0.269
Sex, female	1.34	(1.01—1.78)	**0.046**	1.25	(0.91—1.72)	0.166
Platelet count, × 10^6^/mL	1.46	(1.09—1.96)	**0.011**	1.39	(1.02—1.89)	**0.035**
Lung neoplasm	1.35	(1.01—1.79)	**0.040**	1.15	(0.84—1.59)	0.387
Localised pathology	1.41	(1.09—1.82)	**0.010**	1.44	(1.09—1.90)	**0.011**
Clinician						
Thoracic surgery resident	1.36	(0.53—3.51)	0.523	1.31	(0.47—3.66)	0.611
EM consultant	1.58	(0.78—3.23)	0.205	1.24	(0.54—2.82)	0.611
EM resident	1.97	(0.76—5.07)	0.162	1.19	(0.41—3.41)	0.751
Minimally invasive tube	1.47	(1.04—2.07)	**0.028**	1.27	(0.86—1.89)	0.228
Overnight procedure	1.19	(0.91—1.55)	0.214	1.23	(0.91—1.67)	0.174

*R*^2^ = 0.15. Maximum VIF 1.11. Clinician, thoracic surgery consultant was used as reference. Tube type, trocar was used as reference. EM, emergency medicine

## Discussion

In this observational study, we found that approximately one quarter of patients undergoing tube thoracostomy developed complications. The most frequent of these were malposition/dislodgment, obstruction and pneumothorax. Although no patient in the cohort died as a direct consequence of TT-related complications, the length of hospital stay was significantly longer in those who experienced them. Additionally, patients who developed complications more frequently had neoplastic lung disease as a comorbidity, presented with loculated pleural effusion, had undergone insertion of a small-calibre minimally invasive chest drain, and exhibited elevated platelet counts. Multivariable regression analysis showed that the only independent risk factors for developing complications following emergency TT were the presence of localized pleural pathology, such as loculated pleural effusions, and increased platelet count.

Previous evidence, including studies conducted both in EDs and other hospital settings, has shown a prevalence of chest tube-related complications in line with our current study, ranging between 20 and 35%. [2, 9] TT can be associated with a wide range of complications, but the most frequently reported in the literature and in this study was malposition or dislodgment of the chest drain [3]. While this most frequently impair the functionality of the chest tube and require repositioning, in rare cases it may lead to organ or vascular injuries with potentially fatal consequences [2, 4]. As for infectious complications, we found that the rate of empyema and infection of the insertion site following TT performed in the ED was similar to that reported in studies conducted in more controlled settings [4]. This suggests that, although TTs in the ED may need to be performed under emergent or urgent conditions, appropriate techniques are implemented and sterility is preserved. While in this study no patient died from procedure-related complications, those who experienced complications had significantly longer hospital stays. Similarly, Platnick et al. also reported prolonged hospitalization in patients who developed complications after chest drain placement [4]. Understanding the risk factors underlying TT-related complications may help reduce their occurrence and shorten hospital stays, thereby improving patient care and reducing costs.

This study showed that complications occurred more frequently in patients requiring chest drain placement for loculated pleural effusions. The irregular distribution of fluid in the pleural cavity in loculated effusions complicates appropriate drain positioning and the presence of septations may hinder effective drainage [10]. Loculated effusions, that are most commonly associated with infectious and malignant pathologies, are also usually characterized by floating debris and suspended particles of variable origins (e.g. fibrin clots, cellular debris, pus or malignant cells aggregates), that most likely contribute to chest tube obstructions. In terms of laboratory values on admission, patients who developed complications had higher platelet counts. This finding might appear in contrast with the concept that thrombocytopenia predisposes to bleeding complications. However, in our cohort no TT was performed in patients with platelet count below the recommended safety cut-off considered by many authors > 20 × 10^9^/L [11]. Conversely, it could be hypothesized that the higher platelet count in patients who developed complication may be linked to inflammatory and malignant conditions that, as discussed above, are in turn associated with complicated pleural effusions more prone to cause chest tube obstruction. Previous evidence has shown that higher platelet counts are associated with malignant pleural effusions and worse outcomes [12], and that platelet-derived soluble factors (e.g. platelet factor 4) have been identified in higher concentration within malignant pleural effusions [13], suggesting that the presence and activation of platelets might lead to the formation of aggregates and consequently chest tube obstruction.

The lack of association between operator seniority or specialty and the development of TT-related complications is noteworthy. It is reasonable to assume that less experienced physicians are more prone to procedural errors, posing a higher risk of complications. However, the lack of association between operator experience and complication rate is consistent with the findings reported by Platnick et al. [4] In our institution, this may be explained by the close supervision of trainees by attending physicians during TT placement and a steep learning curve for emergency medicine residents [14]. Several studies have shown that TT performed by non-surgeon specialists is associated with higher complication rates [3, 4, 15]. However, this may reflect variability in emergency medicine training across institutions [4, 14], and the lack of association in this study could reflect recent improvements in the competence of non-surgical operators. Moreover, excluding trauma patients from our study likely removed a cohort in which TT is typically performed under more urgent conditions using larger drains, where operator expertise might play a greater role in safety and success.

The absence of an independent association between the use of minimally invasive chest drains and TT complication is partially in contrast with existing literature, particularly with the results reported by Davies et al. of higher rates of dislodgment and obstruction with small-bore tubes (< 14 Fr) [16]. Although minimally invasive drains are generally associated with less tissue trauma, reduced patient discomfort, and more precise placement, their narrower diameter predisposes them to obstruction by clots or dense material such as pus. Nonetheless, this complication could be effectively prevented through systematic flushing and management of the tube. No independent relationship between procedures performed overnight and TT complications has been found. Previously, it has been suggested a higher risk of complication when TT was performed out of hours [5]. However, the authors reported that out-of-hours procedures were primarily performed by physicians in training in the absence of adequate supervision and support. The consistent supervision by a consultant physician during TT procedure at our institution is possibly responsible of such diverging findings.

This study is affected by a series of limitations. First, the single-centre nature and limited sample size restrained its generalizability. Moreover, the small sample size not only forbade the sub-analysis of risk factors associated with specific TT complications, but also likely limited the statistical power of this study, which was not considered at the time of the study design. Hence, the lack of association between operator seniority, specialty, or minimally invasive chest drains and the development of TT-related complications could potentially represent a type II error. Second, the retrospective design led to a certain degree of missing data, in particular in terms of the chest tube calibre, which could not be investigated in multivariable regression analysis without a further significant loss of statistical power. The retrospective design also prevented the detailed recording of the actual expertise of the resident and consultant physicians who performed the TT investigated in this study. Third, at our institution TT is primarily performed by emergency medicine and thoracic surgery physicians, and thus it was not possible to investigate the impact of other operators on the development of complications. Finally, while it was possible to determine whether in-hospital mortality was related to TT complication, we were unable to specifically identify whether prolonged length of stay was directly related to TT complication or other patients’ factors.

This study shows that performing TT in the ED posed a similar risk of complications than other settings, with the most frequent complications being malposition/dislodgment, obstruction and pneumothorax. The presence of complicated pleural pathology and increased platelet count were identified as independent risk factors for the development of TT complications.

## Data Availability

The datasets generated during and analysed during the current study are available from the corresponding author on reasonable request.
